# GA-Based Membrane Evolutionary Algorithm for Ensemble Clustering

**DOI:** 10.1155/2017/4367342

**Published:** 2017-11-16

**Authors:** Yanhua Wang, Xiyu Liu, Laisheng Xiang

**Affiliations:** School of Management Science and Engineering, Shandong Normal University, Jinan 250014, China

## Abstract

Ensemble clustering can improve the generalization ability of a single clustering algorithm and generate a more robust clustering result by integrating multiple base clusterings, so it becomes the focus of current clustering research. Ensemble clustering aims at finding a consensus partition which agrees as much as possible with base clusterings. Genetic algorithm is a highly parallel, stochastic, and adaptive search algorithm developed from the natural selection and evolutionary mechanism of biology. In this paper, an improved genetic algorithm is designed by improving the coding of chromosome. A new membrane evolutionary algorithm is constructed by using genetic mechanisms as evolution rules and combines with the communication mechanism of cell-like P system. The proposed algorithm is used to optimize the base clusterings and find the optimal chromosome as the final ensemble clustering result. The global optimization ability of the genetic algorithm and the rapid convergence of the membrane system make membrane evolutionary algorithm perform better than several state-of-the-art techniques on six real-world UCI data sets.

## 1. Introduction

Cluster analysis, also known as clustering, is a core technique in machine learning and artificial intelligence [1], which is a process of dividing a data object into subsets, each subset is defined as a cluster, and objects in the same cluster are as similar as possible, yet objects between two clusters are as different as possible.

Ensemble clustering, also known as consensus clustering or cluster aggregation, is simply reconciling clustering result coming from different clustering algorithms [2] or different initialization parameters run in the same algorithm [3]. The purpose of ensemble clustering is to find a consensus result which is as similar as possible to multiple existing base clusterings [4]. Compared with the single clustering algorithm, the clustering ensemble algorithm has higher robustness and stability, and the clustering results are insensitive to noise, isolated points, and sampling changes, so ensemble clustering has become a hotspot of cluster research in recent years. Existing ensemble clustering research methods can be divided into three categories, that is, the median partition based methods [5, 6], the pairwise similarity based methods [7–10], and the graph partitioning based methods [4, 11–13]. Among them, the median partition based methods aim to find a clustering that maximizes the similarity between this clustering and all of the base clusterings which can be viewed as the median point of the median partition [5, 6, 14].

The clustering problem of finding the optimal solution in many base clusterings becomes an optimization problem. Due to the large space of all possible base clusterings, finding the optimal solution is generally infeasible, and genetic algorithm as a classic optimization problem solving method has attracted my attention. Genetic algorithm is a randomized search method which simulates the evolution of biological laws [15]. It has inherent parallelism and global optimization ability. Using probabilistic optimization method, it can automatically obtain and guide the optimization search space and adaptively adjust the search direction [16–18]. The ensemble clustering problem is generally regarded as the median partition problem. In fact, the median partition problem is NP-complete [5]. Genetic algorithm has been proposed to find the approximative solution, in which the base clusterings are represented as chromosomes [5, 19]. In their study, chromosome is defined by base clustering class labels; when the number of data objects is large, the evolutionary efficiency is very low. In this paper, we improve the coding of chromosomes, and then the improved genetic algorithm is combined with membrane computing model for ensemble clustering.

P system, also known as a novel membrane computing model, is a biological computational model inspired by the study of the living cells, initiated by Păun in 1998. It aims to achieve calculation process by simulating the function of living cells, tissues, and organs. Objects in this model, which has complete computing capability, can evolve in a maximal parallelism and distributed manner [20]. It is exactly because of the maximum parallelism of membrane system that realizes multiple cell object concurrent evolution to search the optimal solution, which is similar to the effect of multipopulation evolution, thus making better performance of ensemble clustering. Membrane systems have the same computing power as Turing machines and even do what Turing machines can do more efficient [21, 22]. According to the different organizational structure of the system, the P system is divided into three categories: cell-like P system [23], tissue-like P system [24], and neural-like P system [25]. Among them, the cell-like P system is the first membrane model proposed by scholars, and the research of this P system is also most complete [26–28]. Its basic components include membrane structure, objects, and membrane rules. In the cell-like P system, membranes divide the whole system into different regions in which objects and rules exist; the objects are usually represented by characters or strings of symbols; the rules in each region are used to process the objects in the corresponding membrane. Objects are operated by rules in the membrane in a highly parallel mechanism [29–31], so that the system can make ensemble clustering more efficient.

In this paper, we introduce three genetic operators (selection, crossover, and mutation) of the genetic mechanism to realize the evolution of the chromosome and use the communication mechanism of cell-like P system to realize the sharing of outstanding objects between the membranes; it accelerates the convergence of the algorithm. The proposed algorithm is used to optimize the base clusterings and find the optimal chromosome as the final ensemble clustering result. In Section 2, we give basic concept of ensemble clustering and genetic algorithm and cell-like P system.  Section 3 describes the improved GA-based consensus clustering algorithm.  Section 4 addresses proposed algorithm.  Section 5 shows the result of the experiment and finally we summarized the work in this paper and then plan the future work in Section 6.

## 2. Preliminaries

In this section, we introduce some basic concepts of ensemble clustering, genetic algorithm, and cell-like P system.

### 2.1. Ensemble Clustering

**Figure 1:**
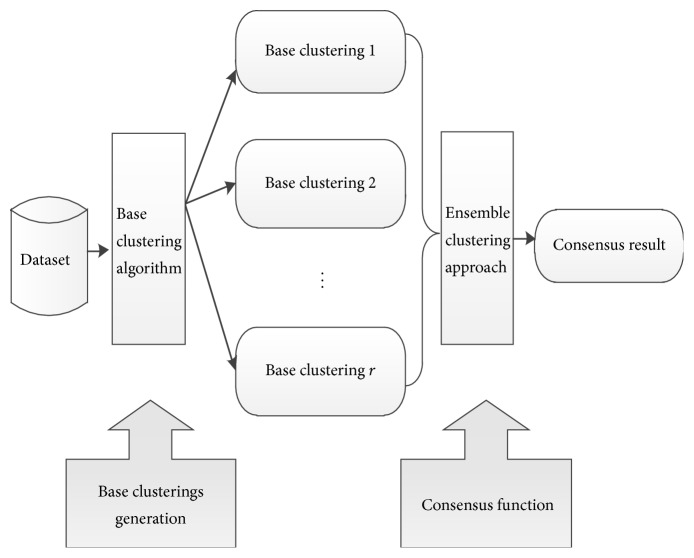
The ensemble clustering process.

Ensemble clustering process is divided into two steps; first we generate a set of different base clusterings and then use consensus function to find a consensus clustering result which agrees as much as possible with existing base clusterings. In order to produce a number of diversified base clusterings, from the perspective of the algorithm, same clustering algorithm can be used with different initialization parameters or the use of different clustering algorithms. From the data set preprocessing point of view, we can choose different attributes or different sample subsets of data sets. The ensemble clustering process is shown as Figure 1.

### 2.2. Genetic Algorithm

Genetic algorithm is one of the intelligent optimization algorithms; it has the advantages of fast search speed, good universality, and global search ability.

The basic steps of genetic algorithm are as follows:

1. Select encoding mode; set the crossover rate, mutation rate, and the evolution generation Gen = 0.
2. The initial population is P(Gen).
3. Calculate the fitness of each chromosome in the population according to the objective function.
4. Gen = Gen + 1.
5. If Gen reaches the set condition, go to step (11); otherwise go to step (6).
6. Two chromosomes are selected from P(Gen − 1), and the probability of selection was proportional to chromosome's fitness.
7. Crossover is performed at a randomly determined point of each pair selected chromosome at a preset hybridization rate.
8. A point is randomly selected from each selected chromosome in accordance with the preselected mutation rate, and the corresponding bit value is changed.
9. The new generated chromosomes and those with high fitness value in P(Gen − 1) are selected for evolution to the next generation P(Gen).
10. If termination condition is not satisfied, go to (3).
11. The chromosome with the highest fitness in the population P(Gen) is the final result, and the algorithm stops.

### 2.3. Cell-Like P System

P system is a distributed, maximal parallelism and nondeterministic computation model; numerous studies [32] have shown that many simple membrane computing models have the same compute power as Turing machines in theory and may even have the potential to go beyond the limitations of Turing machines.

**Figure 2:**
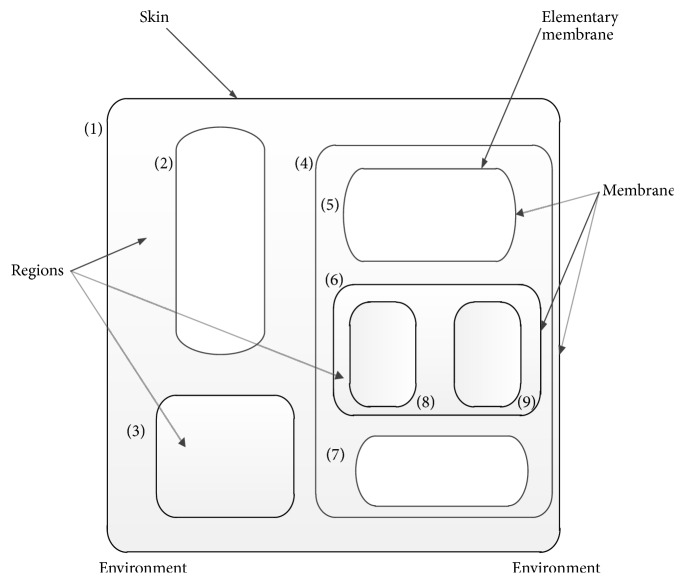
A basic membrane structure.

Cell-like P system is the earliest membrane computing model; three basic elements of the P system are membrane structure, the multiple sets of objects, and evolutionary rules. The data set is represented by strings or characters; objects are controlled by this intramembrane evolution rule and can pass through the membrane. P system is divided into many regions by membranes; the outermost layer of the membrane structure is called skin membrane. A plurality of submembranes is contained in the skin membrane; the basic membrane structure is shown as Figure 2.

A cell-like P system of degree *m* is defined as follows:

(1) $$\begin{array}{c} \prod =\left(\;V,T,C,\mu ,w_{1},\ldots ,w_{m},R_{1},\ldots ,R_{m},\rho_{i},i_{out}\;\right), \end{array}$$

where

1. *V* is an alphabet which includes all the objects of the system.
2. *T*⊆*V* is the output alphabet.
3. *C*⊆*V* − *T* is a set of catalysts whose elements will not change during evolution and do not produce new characters, but they are necessary for some evolutionary rules.
4. *μ* is the membrane structure of degree *m*.
5. *w*_1_,…,*w*_*m*_ ∈ *V* are the multisets of objects in each membrane region *μ*.
6. *R*_*i*_  (1 < *i* < *m*) are the revolutionary rules in membrane *i*.
7. *ρ*_*i*_ is the precedence level of rule *R*_*i*_.
8. *i*_out_ is the output of this P system.

In the cell-like P system, the basic evolutionary rule is the two tuples (*u*, *v*), which can also be expressed as *u* → *v*, *u* is the string of *V*, and *v* = *v*′, or *v* = *v*′*δ*, *v*′ is the string in arbitrary {*a*_here_, *a*_out_, *a*_in_*j*__∣*a* ∈ *V*, 1 < *j* < *m*}, *a*_here_ means the object remains in membrane *i*, *a*_out_ means the object will be sent to the outer membrane, and  *a*_in_*j*__ means the object will be sent to the inner membrane *j*. If the evolutionary rule *R*_*i*_ contains *δ*, this membrane is dissolved after the rule is executed. P system starts with the initial state (represented by the object multiset) and uses the evolutionary rule to process and transport objects to complete the calculation.

## 3. Improved GA-Based Ensemble Clustering Algorithm

### 3.1. Microcluster Based Chromosome Encoding

The fitness function guides the evolution direction of the population. Genetic algorithm is one of the solutions for clustering problem. In the previous studies, in genetic-based ensemble clustering algorithm, the class labels of base clusterings are used as chromosome encoding. When the number of data objects is large, it occupies a lot of space and the efficiency is reduced. In addition, crossover and mutation operations may result in the reassignment of the data points that have been assigned in the same clusters. Specifically, if two objects are divided into the same clusters among all the base clusterings, we consider them fully similar, and they will be considered to be one object that cannot be separated by crossover and mutations operations. So in this paper, we improve the coding of chromosome and proposed the microcluster based chromosome encoding approach.

We introduce the concept of the microcluster for a more compact representation of the base clusterings. Let *X* = {*x*_1_,…, *x*_*i*_} be a date set of *N* objects. We run *r* times basic clustering algorithms to partition *X* to *r* base clusterings ∏ = {*π*_1_, *π*_2_,…, *π*_*r*_}, where *π*_*k*_ is the *k*th base clustering. Let Cls^*k*^(*x*_*i*_) be the cluster in *π*^*k*^ that contains object *x*_*i*_. The objects *x*_*i*_ and *x*_*j*_ are regarded as a microcluster if they are divided into the same cluster for all of the *r* base clusterings; that is, for *k* = 1,…, *r*, Cls^*k*^(*x*_*i*_) = Cls^*k*^(*x*_*j*_).

**Figure 3:**
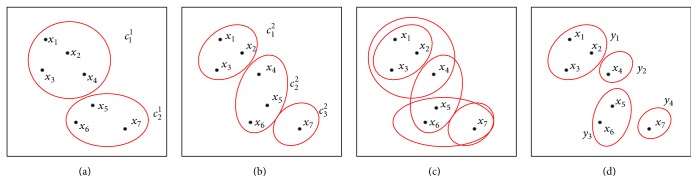
The generation of microcluster.

Given multiple base clusterings, we can obtain a set of *N* nonoverlapping microclusters shown in Figure 3, donated as

(2) $$\begin{array}{c} Y=\left\{\;y_{1},\ldots ,y_{N}\;\right\}. \end{array}$$

In Figure 3, we show the generation process of microcluster, and we use a date set with seven objects as a sample. Two base clusterings *π*_1_ and *π*_2_ are shown in (a) and (b), which contain two clusters and three clusters; we overlap (a) and (b) to get (c); then we generate a set of microclusters in (d). The process of microclusters generation is as shown in Figure 3. *Y* is a set of microclusters, and *y*_*i*_ represents the *i*th microclusters.

In this paper, we use the label of microcluster-based to replace the label of original object to code the chromosome, and a microcluster contains one or many objects that can be regarded as an object in the process of chromosome coding, which can reduce the length of the chromosome and decrease the error caused by mutation and crossover and thereby improve the accuracy of the algorithm. For example, in Figure 3 the two base clusterings are coded with the class label of objects; they are coded as *a* = {1,1, 1,1, 2,2, 2}, *b* = {1,1, 1,2, 2,2, 3} in previous approach; in this paper, we can code them as *a* = {1,1, 2,2}, *b* = {1,2, 2,3}; each base clustering includes four microclusters and coded value represents the cluster labels to which they belong. This method makes the individual coding shorter and thus reduces the search space, and meanwhile the individuals considered to be fully similar in the base clusterings are no longer separated.

### 3.2. Design of Fitness Function

The fitness function guides the evolution direction of the population; the solution of the clustering problem is to find a clustering result that makes the objects in the same cluster have the largest similarity, but the largest difference between two clusters. So in this paper we use a clustering evaluation method OCQ proposed in [33] as fitness function. The definition of OCQ is as follows:

(3) $$\begin{array}{c} OCQ\left(\;\beta \;\right)=1-\left(\;\beta *Cmp+\left(\;1-\beta \;\right)*Sep\;\right), \end{array}$$

where Cmp represents cluster compactness and Sep indicates the cluster's disposability. *β* is the balance coefficient and  0 < *β* < 1, which is used to weight the proportion of the Cmp and Sep, different data sets with different *β* value. Cmp is defined as follows:

(4) $$\begin{array}{c} Cmp=\frac{1}{C}\overset{C}{\underset{i=1}{\sum }}\frac{Dev\left(c_{i}\right)}{Dev\left(D\right)}, \end{array}$$

where *C* is the number of clusters, Dev(*c*_*i*_) is the variance of *c*_*i*_, and Dev(*D*) is the variance of class *D*. Dev(*D*) is defined as follows:

(5) $$\begin{array}{c} Dev\left(\;X\;\right)=\sqrt{\frac{1}{N}\overset{N}{\underset{i=1}{\sum }}d^{2}\left(x_{i},\overset{-}{x}\right)}, \end{array}$$

where *N* is the number of objects in data set *X*, $\overset{-}{x}=\left(1/N\right)\sum_{i=1}^{N}x_{i}$, and *d*(*x*_*i*_, *x*_*j*_) is the distance between *x*_*i*_ and *x*_*j*_. The smaller the value of Dev, the better of the clustering result. Sep is defined as follows:

(6) $$\begin{array}{c} Sep=\frac{1}{C\left(C-1\right)}\overset{C}{\underset{i=1}{\sum }}\;\overset{C}{\underset{j=1,j\neq i}{\sum }}\exp\left[\;-\frac{d^{2}\left(x_{c_{i}},x_{c_{j}}\right)}{2\delta^{2}}\;\right], \end{array}$$

where *δ* is the Gaussian constant, in order to facilitate the calculation, usually 2*δ*^2^ = 1, and *x*_*c*_*i*__, and *x*_*c*_*j*__ are the center of clusters *C*_*i*_ and *C*_*j*_. The larger the value of OCQ, the better of the clustering result.

### 3.3. Elite Selection Function

In this section, we introduce an elite selection strategy to preserve the optimal individual in the evolution of the population. In each generation, a certain number of high fitness chromosomes are selected directly for evolution to the next generation in order to save excellent genes. In addition to the fact that elite strategy improves the evolution efficiency and optimization ability of the proposed algorithm, the ratio *p* of the chromosomes that are directly selected for evolution to the next generation increases linearly with the number of iterations *t*:

(7) $$\begin{array}{c} p=p_{min}+\left(\;p_{max}-p_{min}\;\right)\frac{t-1}{T-1}, \end{array}$$

where *p*_max_ and *p*_min_ are the maximum and minimum selection ratio; when the evolution algebra increases, the proportion of excellent genes in the population also increases, so we design this elite selection function to let the ratio *p* grow with *t*. Experiments show that when the size is 2%~10% of the population, the evolution result is the best, and *p*_max_ and *p*_min_ are set as 0.1 and 0.02, respectively.

## 4. The Proposed GA-Based Membrane Evolutionary Algorithm

### 4.1. The Evolution Rules and the Communication Rules of Cell-Like P System

In cell-like P system, membrane rules mainly include two types of rules, evolutionary rules and communication rules. Evolutionary rules are used to promote the evolution of chromosome. Communication rules are used to communication and share information between two regions.

In this paper, the evolutionary rules contain *K*-means rules, AL, SL, and CL rules [14], selection rules, crossover rules, and mutation rules.

*K*-means rules are used to generate the base clusterings; the detailed description of *K*-means rules is as follows.

Given a data set *x*_1_,*x*_2_,…, *x*_*n*_, and a set of center of cluster *m*_1_,*m*_2_,…, *m*_*k*_, if the distance between *x*_*i*_ and *m*_*j*_ is less than the distance between *x*_*i*_ and *m*_*i*_, the object *x*_*i*_ will be reassigned to *C*_*j*_:

(8) $$\begin{array}{c} \left|\;x_{i}-m_{j}\;\right|<\left|\;x_{i}-m_{p}\;\right|,\;p,j=1,\ldots ,k,\;\;i\neq j. \end{array}$$

When all the points are assigned to the corresponding clusters, the new center of cluster corresponding to each cluster is the average value of the points in this cluster:

(9) $$\begin{array}{c} m_{j}^{*}=\frac{1}{n_{j}}\underset{x_{i}\in C_{j}}{\sum }x_{i},\;j=1,\ldots ,k, \end{array}$$

where *m*_*j*_^*∗*^  is  the center of the new cluster *C*_*j*_ and *n*_*j*_ is the number of objects belonging to *C*_*j*_.

For AL, SL, and CL rules, two partitions with the highest similarity are merged into a new bigger partition and thus the number of objects will finally reduce to one. The similarity of two partitions will be computed by the mentioned three rules. Let *P*^(*t*)^ = {*P*_1_^(*t*)^,…, *P*_|*P*^(*t*)^|_^(*t*)^} be the set of merged partition in the *t*-step for *t* = 1,2,…, *N*. *N* is the number of objects of date set. |*P*^(*t*)^| represents the number of partitions in *P*^(*t*)^. Each partition contains one or more microclusters. Let *y*_*i*_ represent a microcluster; we write *y*_*i*_ ∈ *P*_*j*_^(*t*)^ if microcluster belongs to *P*_*j*_^(*t*)^. Let *S*^(*t*)^ = {*s*_*ij*_^(*t*)^}_|*P*^(*t*)^|*∗*|*P*^(*t*)^|_; the similarity matrix for *P*^(*t*)^, AL, SL, and CL rules can be operated as follows:

(10) sijt=1Pit·pjt∑yk∈Pit,yl∈PjtSimklIf  Method  AL,∑yk∈Pit,yl∈PjtSimklIf  Method  CL,maxyk∈Pit,yl∈Pjt⁡SimklIf  Method  SL,

where Sim_*kl*_ is the Cosine similarity and |*P*_*i*_^(*t*)^| is the number of microclusters of *P*_*i*_^(*t*)^.

Selection rules imitate the nature laws of natural selection, which are used to select objects from population to evolution to the next generation. In this paper, we calculate the fitness value of each chromosome, and then the selection probability of each chromosome is obtained based on the fitness value. Each chromosome is selected to do crossover and mutation to improve the fitness. And then a certain percentage of chromosomes with high fitness are chosen as candidate set evolution to the next generation. We use the usual rotating wheel method to define selection rule; the selection probability formula is as follows:

(11) $$\begin{array}{c} \theta \left(\;i\;\right)=\frac{f\left(i\right)}{\sum_{i=1}^{r}f\left(i\right)}, \end{array}$$

where *r* is the number of the chromosomes and *f*(*i*) is the fitness value of each individual.

In the evolutionary process, the algorithm often falls into the local optimum, crossover rate and mutation rate are increased to improve the global convergence [34], and the crossover function is as follows:

(12) $$\begin{array}{c} P_{c}\left(\;Gen\;\right)\left\{\begin{array}{cc} P_{c_{temp}}, & P_{c_{temp}}>P_{c_{min}}\\ P_{c_{min}}, & others, \end{array}\right. \end{array}$$

where *P*_*c*_temp__ = *P*_*c*_max__*∗*2^(−Gen/MaxGen)^, *P*_*c*_max__ is predefined maximum crossover rate, and  *P*_*c*_min__ is the minimum crossover rate.

The mutation function is as follows:

(13) $$\begin{array}{c} P_{m}\left(\;Gen\;\right)=\left\{\begin{array}{cc} P_{m_{temp}}, & P_{m_{temp}}>P_{m_{min}}\\ P_{m_{min}}, & others, \end{array}\right. \end{array}$$

where *P*_*m*_(Gen) = 1/(1 + Gen/MaxGen)*∗P*_*m*_max__,*P*_*m*_max__, and *P*_*m*_min__ are predefined maximum mutation rate and minimum mutation rate.

The crossover rule uses the single-point crossover in which the intersection is according to the crossover probability (12). The single-point mutation is used to realize the mutations of objects and produce new individuals. Since the mutation operation has a certain degree of blindness, we set the mutation probability very small, and the mutation probability is calculated as (13). If *m* is a mutation point determined by the mutation function *p*_*m*_, its value becomes *m*′ = random(1, *C*), which means a random positive integer between (1, *C*), and *C* is the maximum value of the present mutation individual.

*Communication Rules*. Communication rules enable the exchange of information between two membranes, share excellent objects, and promote the evolution of the object set in each membrane. The form of the communication rule is as follows:

(14) $$\begin{array}{c} \left(\;i,\frac{\mu }{v},j\;\right). \end{array}$$

This communication rule means object *μ* in membrane *i* is exchanged with the object *v* in membrane *j*; if *v* = *λ* means *v* is null, *μ* is transported to *v*, and vice versa. In this paper, we define a copy of object *μ* that still remains in membrane *i* after *μ* is transported to *v*.

### 4.2. Description of the Proposed GA-Based Membrane Evolutionary Algorithm

**Figure 4:**
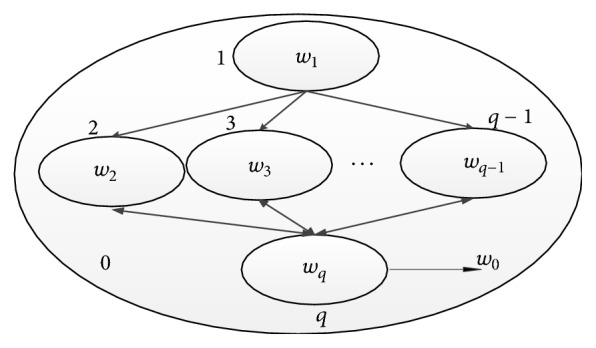
The membrane structure for the GMEAEC.

In this section, we design the membrane structure for proposed algorithm which is shortly called GMEAEC and descript the algorithm process. The membrane structure is as shown in Figure 4.

This cell-like P system is defined as follows:

(15) $$\begin{array}{c} \prod =\left(\;w_{1},\ldots ,w_{q},R_{1},\ldots ,R_{q-1},R_{q},i_{out}\;\right), \end{array}$$

where

1. *w*_1_ represents the initial objects in membrane 1; initial objects are the data to be clustered. *w*_2_,…, *w*_*q*−1_, are the base clusterings randomly selected from membrane 1, *w*_*q*_ are elite individuals selected from *q* − 2 subpopulations according to the probability (7), and  *w*_0_ is the best chromosomes in each generation preserved in membrane 0.
2. *R*_1_,…, *R*_*q*−1_ are the evolution rules in membrane 1,…, *q* − 1, *R*_1_ are the evolution rules which are used to generate base clusterings including *K*-means rules, and AL, CL, and SL rules, *R*_2_,…, *R*_*q*−1_, include select rule, crossover rule, mutation rule, and communication rule in membrane 2,…, *q* − 1, which are used to achieve the evolution of the population, while *R*_*q*_ is the rule in membrane *q* that is the communication rule.
3. *i*_out_ is the output result in membrane 0.

The description of the algorithm process is as follows:

1. Run base clusterings algorithm *r* times in membrane 1 to construct a pool of base clusterings and then generate microcluster representation.
2. Randomly select the same number of base clusterings from membrane 1 to membrane 2,…, *q* − 1, respectively, to construct multiple population.
3. Initialize the population; each chromosome is coded by a base clustering represented by the microcluster-based label.
4. Calculate the fitness of the individuals according to the fitness function.
5. Transport *m*-elite individuals of each subpopulation to membrane *q* to construct (*q* − 2)*m* elite individuals and simultaneously original populations keep a copy.
6. Use selection rules to select the chromosomes according to the predefined probability, and use crossover rule and mutation rule to promote chromosomes evolution; the population in each membrane evolves in parallel.
7. Sort the fitness of the (*q* − 2)  *m* chromosomes in the membrane *q* and then select the top-*m* chromosomes and transport them to membrane 2,…, *q* − 1 to replace the *m* low fitness chromosomes.
8. Transport the best chromosome to membrane 0; if its fitness value is larger than the present one, replace it, or else abandon it.
9. If the condition is satisfied, the algorithm ends, and we obtain the highest fitness chromosome; then map microclusters back to objects and output the objects in the membrane 0, or else repeat (4)–(9).

**Figure 5:**
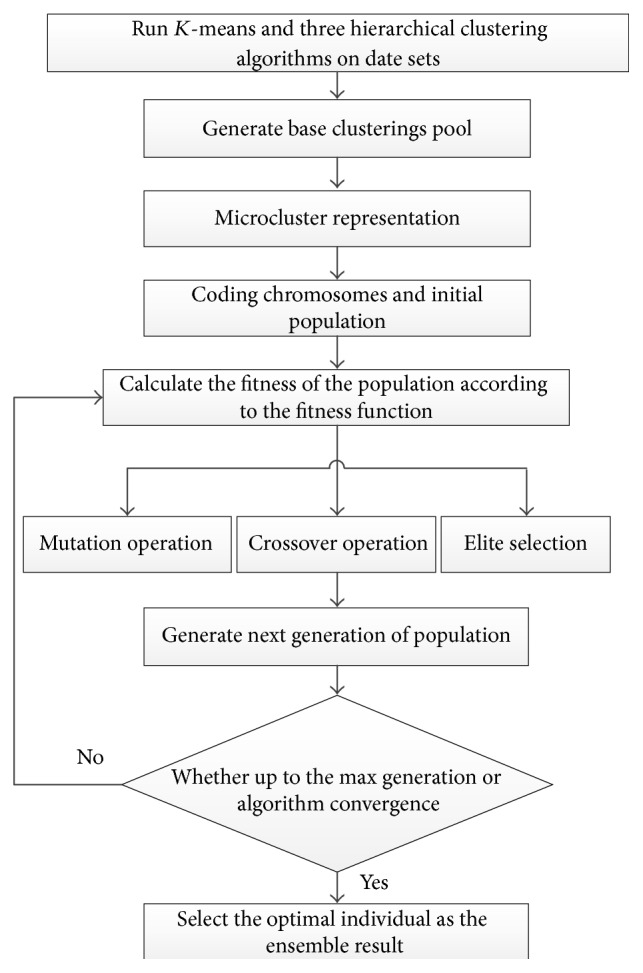
Flow diagram of the proposed approach.

The overall process of our approach is shown in Figure 5. We first use *K*-means and three agglomerative methods to generate base clusterings pool, and then we assign the data objects to the microcluster, after that we code the chromosome with label of microcluster-based introduced in Section 3.1. The evolutionary mechanism of GA will find the final ensemble result.

The membrane evolutionary algorithm takes the advantage of the maximum parallelism of membrane systems and global search optimization ability of genetic algorithm; in the base clusterings generation step, we use four algorithms combined with different initial parameters to obtain diversified base clustering which make the ensemble result share the information of many single clustering results and integrate them to get a better ensemble clustering result than any one of them. In the ensemble clustering step, the result is obtained by the membrane evolutionary algorithm which uses the improved genetic algorithm; the improved encoding of the chromosome regards the objects assigned in the same clusters for all base clustering as a microcluster, so that they will not be separated by crossover and mutation operation which increases the accuracy of the clustering. In addition, the elite selection strategy and parallelism of membrane systems make the *m*-elite chromosomes be generated synchronously in each membrane and the *m*-elite chromosomes among them are transported to all membranes to guide the evolution of the next generation. All of the above make the GMEAEC performs better than other algorithms.

### 4.3. Time Complexity Analysis

In this section, the time cost in the worst case of GMEAEC is analyzed. In the base clustering generation step, we put the objects in membrane 1 and use *K*-means and three agglomerative clustering methods with different initial parameters to generate base clusterings. Let dataset *D* have *n* records; each record has *m* attributes; we partition the date set to *k* clusters; the computational complexity of *K*-means is *O*(*knthm*), where *t* is the number of iterations for the convergence of *K*-mean clustering and *h* is the number of base clusterings generated by *K*-means. The computational complexity of three agglomerative methods is *O*(*h*(*n* − *k*)*mnn*), and *h* is the number of base clusterings generated by each agglomerative method. After generating base clusterings pool, we can compute microclusters, and the complexity of the microclusters generation is *O*(*n*); the complexity of the integration step is *O*(MaxGen*∗hknm*), where MaxGen is the number of iterations for convergence of genetic algorithm. As a result, the complexity of the base clustering generation is *O*(*knthm*) + *O*(*h*(*n* − *k*)*mnn*), and the complexity of the ensemble clustering step is *O*(MaxGen*∗hknm*) + *O*(*n*) = *O*(MaxGen*∗hknm*).

## 5. Experiment Analysis

### 5.1. Experimental Setup

*Experimental Data*. We use six real-world data sets of UC Irvine Machine Learning Repository [35] in our experiment.  Table 1 shows some important characteristics of these data sets.

*Validation Measure*. It is used to measure the accuracy of the proposed algorithm; in this paper, we use normalized rand index (*R*_*n*_) [36] since the cluster label of all data sets is known. Its value usually ranges between [0, 1]. The higher value means the high accuracy of the clustering result.

*Base Clusterings Generation*. It has been shown that ensemble clustering will be more effective when the base clusterings errors are different; that is, diversity among the base clusterings will enhance the ensemble result. A single clustering algorithm over many iterations usually generates similar result, so for each dataset we use *K*-means and three agglomerative clustering methods, namely, average-linkage (AL), complete-linkage (CL), and single-linkage (SL) to generate base clusterings pool, with initial number of clusters *k* randomly within [*K*, *b*]; *K* is the true number of clusters = min$\left\{\sqrt{N}/2\right\}$, and *N* is the number of the data sets. By running *K*-means and AL, CL, and SL 50 times, respectively, a pool of 200 base clusterings is obtained for each benchmark dataset, for each run of the proposed algorithm and comparison ensemble algorithm we randomly select *M* base clusterings for ensemble. To rule out the factor of getting lucky occasionally, for each *M* we repeat selection many times for each experiment and get the average performance of all ensemble methods. Unless specially mentioned, the ensemble size is *M* = 10 in our experiment.

*Parameter Setting*. The maximum iterate times of the proposed algorithm are set according to the dataset size. The crossover rate and mutation rate are set as follows: *P*_*c*_max__ and *P*_*c*_min__ are 0.3 and 0.1 *P*_*m*_max__ and *P*_*m*_min__ are 0.09 and 0.01. We design the crossover rate and mutation rate associated with the evolution algebra to improve the global convergence of the proposed algorithm. The number of the membranes is *q* = 12, among which membrane 0 is used for saving the optimal solution and membrane 1 is used to generate base clustering pool, membrane *q* is used for preserving the better individual in each population, and other membranes are used for the evolution of individuals in a parallel way; among them the top-*m* individuals with high fitness will directly evolve to the next generation. Evolution generation is various in different data sets for the best result.

### 5.2. Comparison against Base Clusterings

**Figure 6:**
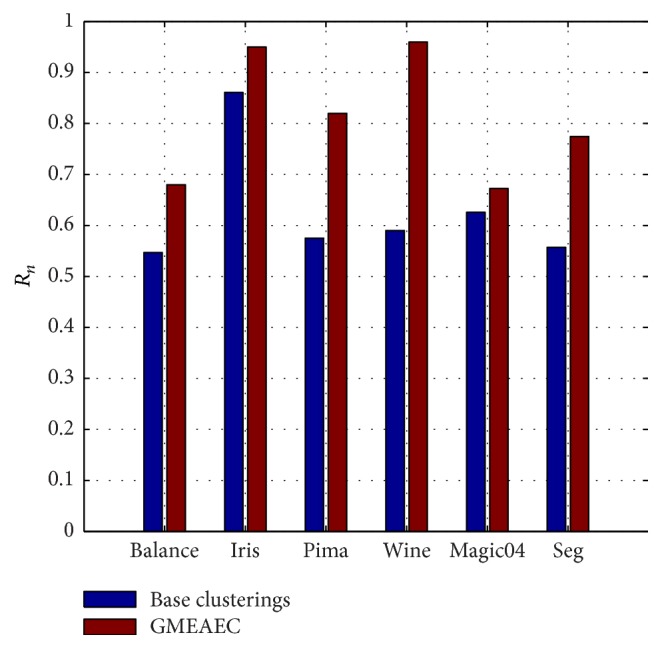
GMEAEC versus base clusterings.

The purpose of the ensemble clustering is to generate a more accuracy and robust clustering result than base clusterings algorithm by integrating multiple base clusterings results to a consensus one; in this section, we compare our proposed algorithm GAEAEC against the base clusterings to prove the effectiveness of the algorithm. The average value of *R*_*n*_ scores is obtained over 100 times runs for each algorithm. As shown in Figure 6, the proposed GMEAEC algorithm outperforms base clusterings algorithms on all of the given data sets.

### 5.3. Comparison against Other Ensemble Clustering Approaches

In this section, we evaluate the effectiveness and robustness of the proposed algorithm by comparing it with five other ensemble clustering approaches, five types of ensemble clustering method, namely, *K*-means based consensus clustering (KCC) [37]; a GA-based ensemble clustering algorithm [19] which is shortly called CEGA; and three graph partitioning algorithms, CSPA, HGPA, and MCLA [4] which are employed for the comparison purpose. KCC is a method which transforms the consensus clustering to *K*-means clustering by the contingency matrix and binary data set. CEGA is a GA-based ensemble clustering method which encodes the chromosome with the class label of the base clusterings. CSPA is one of the most primitive ensemble clustering methods; if the objects are divided into the same cluster for all base clusterings, then they are considered to be completely similar; if not they are dissimilar, and the similarity of two objects is defied by the probability of dividing into the same clusters. Based on the above description, the entire *n∗n* matrix *S* can be computed in one sparse matrix multiplication *S* = *T*_*ij*_/*r*, *r* is the number of base clusterings, *T*_*ij*_ is the times of objects *i*, and *j* belongs to the same clusters. The graph partitioning method METIS algorithm [38] is used to partition the similarity graph (vertex = object, edge weight = similarity). HGPA is a hypergraph partitioning algorithm, each data is regarded as vertices with the same weight, and each cluster is considered as a hyperedge. The ensemble clustering is converted into a hypergraph partitioning by cutting the graph into *k* partitions with the minimal cut. The idea of MCLA is to group the hyperedges which is represented by clusters and divide the object to the hyperedges in which it participates most times.

**Figure 7:**
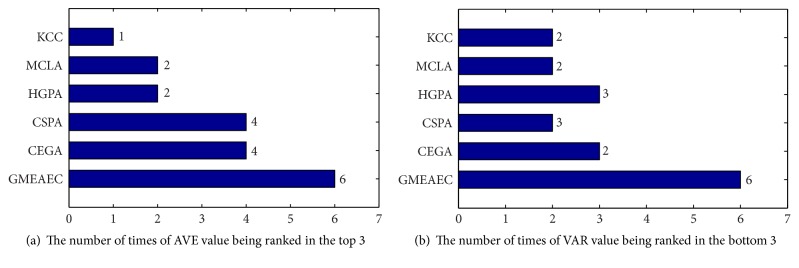
The number of times each approach is ranked in the top (bottom) 3 across Table 2.

We run the proposed GMEAEC algorithm and another ensemble clustering algorithm 100 times on each data set; for each run, the base clusterings are randomly selected from the base clusterings pool, and the number of the base clusterings is preset. More detail about it and parameter setting is descripted in Section 5.1. We show the statistics of the max, min, average (ave), and variance (var) of *R*_*n*_ value in Table 2; we use two criteria, average value and variance, to evaluate the accuracy and the robustness of the proposed algorithm. We can see from Table 2 that the top 3 highest scores of average value and the bottom 3 scores of variance are highlighted in bold. The proposed algorithm achieves the highest scores for balance, pima, and wine datasets, both average value and maximum value in terms of *R*_*n*_ for 100 runs, while the variance values for wine and magic04 datasets are the lowest. To compare the performance of these approaches in a clear way, Figure 7(a) shows the number of each approach to be ranked in the top 3 of the average value which indicates the accuracy of the algorithm.  Figure 7(b) shows the number of each approach to be ranked in the bottom 3 of the variance value which illustrates the stability and robustness of the algorithm. The proposed algorithm achieves the overall best performance in both clustering accuracy stability and robustness compared to other ensemble clustering approaches for all the datasets.

### 5.4. Robustness to Ensemble Size *M*

**Figure 8:**
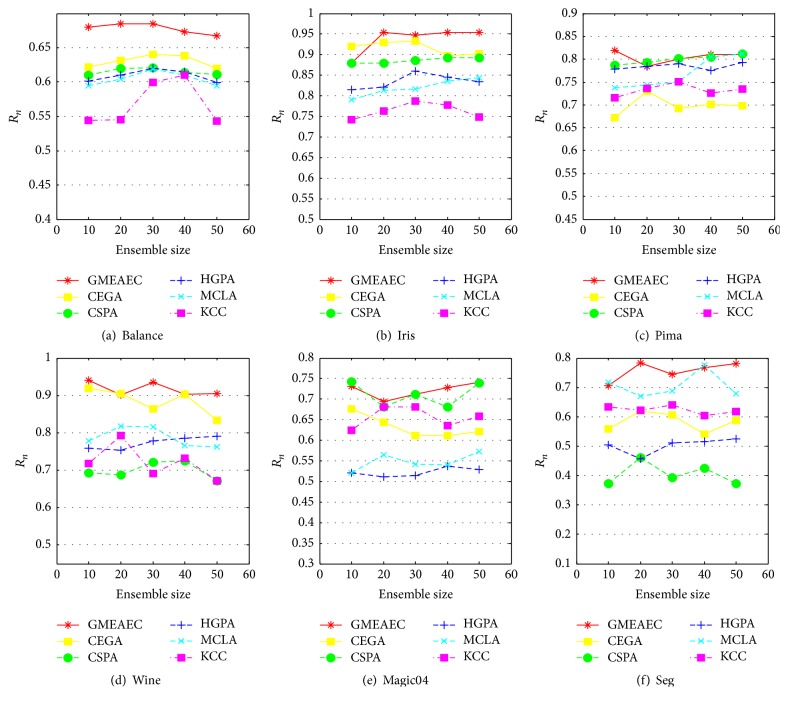
The average performances over 10 runs on different methods by varying ensemble sizes *M*.

In this section, we further evaluate the robustness of GMEAEC by varying the size of base clusterings. For each dataset, we, respectively, select 10, 20, 30, 40, and 50 base clusterings for clustering ensemble. For each *M*, we run the GMEAEC and other ensemble clustering algorithms for 10 times and report the average scores in Figure 8. We can see from Figure 8 that the GMEAEC performance is nearly consistently the best for all ensemble sizes *M* and significantly better than other ensemble methods for all the dataset. Especially for balance dataset, the GMEAEC appears obviously superior on various ensemble sizes than other methods, which demonstrates the advantage of our method in robustness for all dataset and ensemble size.

## 6. Concluding Remarks

In this paper, we improve coding of chromosomes in the previous study; a microcluster-based chromosome encoding is designed to improve the accuracy of ensemble clustering. The improved genetic algorithm contains select rule, crossover rules, and mutation rules. These rules are used as evolution rules to combine with the communication mechanism of cell-like P system. This novel GA-based membrane evolution algorithm is proposed for ensemble clustering. The global convergence of the proposed algorithm and parallel computing ability of cell-like P system make it show better performance in six real-world data sets. In the future, we will combine the GA with other evolutionary algorithms and other membrane systems to improve accuracy and efficiency of ensemble clustering.

## Figures and Tables

**Table 1:** Some characteristics of data sets.

Data sets	Source	Objects	Attributes	Classes
Balance	UCI	625	4	2
Iris	UCI	150	4	3
Pima	UCI	768	8	2
Wine	UCI	178	13	3
Magic04	UCI	19020	10	2
Segmentation	UCI	2100	19	7

**Table 2:** Average performances (in terms of *R*_*n*_) over 100 runs by different ensemble clustering methods (the three highest scores of AVE and the three lowest scores of Var in each column are highlighted in bold).

Method	Balance				Iris				Pima			
	MAX	MIN	AVE	VAR	MAX	MIN	AVE	VAR	MAX	MIN	AVE	VAR
GMEAEC	**0.723**	0.621	**0.679**	**0.00133**	0.917	0.877	**0.881**	**0.00099**	**0.833**	0.733	**0.820**	**0.00254**
CEGA	0.699	0.542	**0.622**	**0.00544**	**0.937**	0.755	**0.920**	0.00756	0.725	0.633	0.676	**0.00375**
CSPA	0.711	0.520	**0.610**	0.00989	0.920	0.794	**0.879**	0.00482	0.820	0.712	**0.787**	0.00543
HGPA	0.655	0.578	**0.610**	**0.00067**	0.842	0.702	0.815	**0.00082**	0.830	0.648	**0.778**	0.01211
MCLA	0.633	0.456	0.594	0.01012	0.830	0.768	0.791	**0.00101**	0.820	0.662	0.738	0.00378
KCC	0.694	0.377	0.544	0.01982	0.878	0.544	0.742	0.01351	0.735	0.698	0.716	**0.00012**

Method	Wine				Magic04				Seg			
	MAX	MIN	AVE	VAR	MAX	MIN	AVE	VAR	MAX	MIN	AVE	VAR

GMEAEC	**0.952**	0.878	**0.941**	**0.00134**	0.783	0.655	**0.731**	**0.00134**	0.751	0.615	**0.707**	**0.00589**
CEGA	0.930	0.840	**0.920**	**0.00252**	0.712	0.542	**0.677**	0.00942	0.659	0.421	0.558	0.00983
CSPA	0.723	0.553	0.693	**0.00142**	**0.824**	0.554	**0.743**	0.01564	0.456	0.235	0.373	**0.00873**
HGPA	0.830	0.662	0.759	0.00756	0.577	0.432	0.520	**0.00546**	0.658	0.423	0.504	0.01425
MCLA	0.879	0.320	**0.776**	0.09844	0.654	0.344	0.526	0.02121	**0.778**	0.684	**0.717**	**0.00178**
KCC	0.886	0.226	0.717	0.11254	0.756	0.498	0.624	**0.00899**	0.755	0.524	**0.633**	0.00997
